# Personalized Risk Stratification of Residual Histologic Activity in IBD Using Circulating Cytokines

**DOI:** 10.3390/jpm16050275

**Published:** 2026-05-21

**Authors:** Nikolaos Martinos, Christos Kroupis, Marilena Stamouli, Andreas C. Lazaris, Georgia-Eleni Thomopoulou

**Affiliations:** 1 Gastroenterology Department, Naval Hospital of Athens, 11521 Athens, Greece; 2 Department of Clinical Biochemistry, Attikon University General Hospital, National and Kapodistrian University of Athens, 12462 Athens, Greece; 3 Department of Biochemistry, Athens Naval and Veterans Hospital, 11521 Athens, Greece; marilena_stamouli@yahoo.com; 4 First Department of Pathology, School of Medicine, National and Kapodistrian University of Athens, 11527 Athens, Greece; alazaris@med.uoa.gr; 5 Cytopathology Department, Attikon University General Hospital, National and Kapodistrian University of Athens, 12462 Athens, Greece; gthomop@med.uoa.gr

**Keywords:** inflammatory bowel disease, endoscopic remission, cytokines, interleukin-10, interleukin-23, risk stratification, biomarkers

## Abstract

**Background:** Persistent histologic inflammation may remain present in patients with inflammatory bowel disease (IBD) despite endoscopic remission. This study evaluated a clinically interpretable cytokine-based framework for risk stratification of residual histologic activity. **Methods:** In this prospective cohort study, 59 patients with IBD undergoing colonoscopy were included. Primary analyses were restricted to patients with ulcerative colitis (UC) in endoscopic remission (*n* = 31). Histologic activity was assessed using the Geboes score. Serum interleukin-10 (IL-10), interleukin-23 (IL-23), and C-reactive protein (CRP) were measured prior to endoscopy. Receiver operating characteristic (ROC) analysis and ROC-derived thresholds were used to evaluate biomarker performance and construct a cytokine-based risk stratification framework. **Results:** Among patients with UC in endoscopic remission, 14/31 (45.2%) demonstrated persistent histologic activity. IL-10 showed the strongest discriminatory performance for histologic activity (AUC 0.850), with a threshold < 3.9 pg/mL associated with sensitivity of 84.6% and specificity of 77.8%. Similar performance was observed using raw assay-reported IL-10 values (AUC 0.906). IL-23 showed limited overall discrimination (AUC 0.615). A combined IL-10/IL-23 framework stratified patients into progressively higher-risk subgroups, with histologic activity observed in 1/14 patients (7.1%) in the low-risk subgroup, 1/2 patients (50.0%) in the Intermediate A subgroup, 9/12 patients (75.0%) in the Intermediate B subgroup, and 3/3 patients (100%) in the high-risk subgroup (*p* < 0.001), although estimates for smaller subgroups should be interpreted cautiously. Reduced IL-10 levels were independently associated with histologic activity, whereas IL-23 primarily refined subgroup classification without substantially improving discrimination. **Conclusions:** An exploratory cytokine-based framework incorporating IL-10 and IL-23 may support risk stratification of residual histologic activity in UC during endoscopic remission. Larger multicenter studies are required to validate these findings and define their clinical utility.

## 1. Introduction

Inflammatory bowel disease (IBD), including ulcerative colitis and Crohn’s disease, is a chronic relapsing condition characterized by intestinal inflammation resulting from dysregulated immune activity [1]. Contemporary management has evolved toward a treat-to-target approach, emphasizing objective markers of disease control. Endoscopic remission has therefore become a central therapeutic goal and is associated with improved long-term outcomes.

However, accumulating evidence indicates that endoscopic remission does not consistently reflect complete resolution of mucosal inflammation. Persistent histologic inflammation may remain present in a significant subset of patients despite apparent macroscopic remission, a phenomenon referred to as endoscopic–histologic discordance [2,3]. Ongoing microscopic inflammatory activity has been identified as a predictor of future disease relapse, disease progression, and the need for treatment escalation, highlighting an important gap between conventional treatment targets and underlying disease biology.

While the clinical significance of histologic activity is increasingly recognized, its integration into routine disease monitoring remains limited. In particular, there is a need for non-invasive approaches capable of identifying patients with residual microscopic inflammation within the endoscopic remission population. Conventional systemic biomarkers, such as C-reactive protein (CRP), provide only indirect and often insensitive estimates of inflammatory burden, especially in patients with apparently quiescent disease [4,5,6,7,8].

Importantly, established non-invasive biomarkers such as fecal calprotectin are widely used in clinical practice and have demonstrated a strong correlation with mucosal inflammation. However, fecal calprotectin primarily reflects neutrophil-driven intestinal inflammation and may not fully capture the complexity of immune regulatory mechanisms, particularly in patients with endoscopic remission. Therefore, there remains a need to explore complementary biomarker approaches that may provide additional biological insight beyond current standard-of-care tools.

Circulating cytokines have been investigated as potential correlates of mucosal immune activity, reflecting the balance between regulatory and pro-inflammatory pathways. Interleukin-10 (IL-10), a key anti-inflammatory cytokine, plays a central role in maintaining intestinal immune tolerance, whereas interleukin-23 (IL-23) promotes sustained inflammatory responses through activation of pathogenic T-cell pathways. Although prior studies have described associations between cytokine profiles and histologic disease activity, the translation of these biological signals into clinically actionable frameworks remains an unmet need [9,10,11,12,13].

From a personalized medicine perspective, the identification of biologically distinct patient subsets within clinically similar states represents a critical step toward individualized disease monitoring and management [14,15,16,17]. Rather than focusing solely on group-level associations, an emerging challenge lies in operationalizing biomarker signals into clinically interpretable models that can potentially support individualized clinical decision-making [18,19,20,21].

In this context, combining regulatory and pro-inflammatory cytokine signals may enable the development of simplified, risk-based frameworks capable of identifying patients at low, intermediate, or high risk of residual histologic activity. Such approaches may support a shift from uniform monitoring strategies toward risk-adapted clinical decision-making [22,23].

In the present cross-sectional analysis nested within a prospectively established cohort, we aimed to develop and evaluate a cytokine-based stratification approach for detecting residual microscopic inflammation in patients with IBD who are in endoscopic remission. Specifically, we investigated whether IL-10 and IL-23 could be integrated into a clinically interpretable model that enables patient-level risk classification, with the goal of supporting individualized consideration regarding histologic assessment and therapeutic evaluation [24,25,26,27]. This approach should be considered exploratory and hypothesis-generating, and aims to provide a preliminary framework rather than a definitive clinical tool. This analysis builds upon prior investigations of cytokine–disease relationships by focusing specifically on the development of a clinically applicable stratification framework.

## 2. Materials and Methods

### 2.1. Study Design and Participants

The study was based on a prospectively established single-center observational cohort from the Hepato-Gastroenterology Unit of the Naval Hospital of Athens, Greece. Clinical, endoscopic, histologic, and biomarker data were collected using predefined standardized procedures prior to outcome assessment.

Consecutive adult patients (≥18 years) with a confirmed diagnosis of inflammatory bowel disease (IBD), including ulcerative colitis (UC) and Crohn’s disease (CD), who underwent clinically indicated colonoscopy between March 2025 and August 2025 were considered eligible. Patient identification for potential eligibility was initiated in March 2025 based on routine clinical scheduling logs. No study-specific data collection, data extraction, or analysis was performed prior to institutional ethics approval. Formal enrollment and prospective data collection commenced only after approval was obtained in April 2025.

The overall cohort was originally designed to investigate circulating cytokines in relation to disease activity. However, the present analysis was prospectively defined with a distinct objective, focusing on the development of a cytokine-based framework for patient-level risk stratification of residual histologic activity within the endoscopic remission population. Accordingly, the current study represents a cross-sectional analytical approach embedded within a prospective cohort, rather than a retrospective analysis.

Diagnosis of IBD was established according to internationally recognized clinical, endoscopic, radiologic, and histopathologic criteria. Included patients underwent colonoscopy as part of routine monitoring or clinical evaluation while receiving standard-of-care treatment. Current treatment exposure, including biologic therapy, immunomodulators, corticosteroids, and 5-aminosalicylic acid (5-ASA), was recorded at the time of inclusion and subsequently explored across phenotype groups as a potential source of cytokine-related confounding. For exploratory treatment-stratified analyses, advanced biologic therapy and combination therapy were categorized as biologic therapy, whereas 5-aminosalicylic acid (5-ASA), conventional non-biologic therapy, and no treatment were categorized as non-biologic therapy.

Predefined exclusion criteria included age <18 years, pregnancy, active infection at the time of assessment, known hereditary disorders, history of or active malignancy, severe organ dysfunction, and antibiotic use within four weeks prior to inclusion. To preserve the biological interpretability of cytokine measurements, patients receiving monoclonal antibodies targeting the interleukin-23 (IL-23) pathway, or with exposure within the preceding 12 months, were excluded due to potential pharmacologic interference with circulating cytokine levels.

Patients with incomplete endoscopic, histologic, or biomarker data were excluded. The final analytical cohort included only patients with complete IBD-related data (*n* = 59). Given the observed imbalance in disease subtype distribution across endoscopic–histologic phenotypes and the limited number of Crohn’s disease cases with endoscopic–histologic discordance, the primary analytical framework was prospectively restricted to patients with ulcerative colitis (UC) in endoscopic remission. Exploratory pooled UC/CD analyses were additionally performed as sensitivity analyses.

Importantly, healthy controls enrolled in the broader cohort were not included in this analysis, as the primary objective was not case–control comparison but rather within-disease stratification across clinically relevant endoscopic–histologic phenotypes. This approach aligns with a personalized medicine framework, emphasizing heterogeneity within clinically defined disease states.

Given the exploratory character of the study and the limited prior evidence available for effect size estimation in cytokine-based risk stratification of endoscopic–histologic discordance, a predefined sample size calculation was not performed. Consequently, the relatively limited sample size, particularly within subgroup analyses, may reduce statistical power and limit the precision and generalizability of the findings. Therefore, the findings should be interpreted as preliminary and require validation in larger cohorts.

### 2.2. Ethical Approval

The study was conducted in accordance with the ethical principles of the Declaration of Helsinki (1975, revised in 2013). The study protocol was reviewed and approved by the Institutional Review Board of the Naval Hospital of Athens (protocol code 3366; approval date: 11 April 2025) and by the Institutional Review Board of Attikon University Hospital (protocol code 219; approval date: 8 April 2025).

All participants provided written informed consent prior to inclusion in the study, including consent for the use of clinical data and biological samples for research purposes.

### 2.3. Endoscopic and Histologic Assessment

Endoscopic evaluation was performed as part of routine clinical management using validated disease-specific scoring systems. In patients with ulcerative colitis (UC), mucosal activity was assessed using the Mayo endoscopic subscore, with remission defined as a score ≤ 1. In Crohn’s disease (CD), endoscopic severity was quantified using the Simple Endoscopic Score for Crohn’s Disease (SES-CD), with endoscopic remission defined as SES-CD ≤ 2.

During colonoscopy, mucosal biopsies were obtained according to standard clinical protocols from multiple intestinal segments. Sampling included both visually normal and inflamed mucosa, when present, to capture potential spatial heterogeneity of microscopic inflammation.

Collected tissue samples were fixed in formalin and processed using routine histopathologic techniques. Histologic evaluation was conducted by experienced gastrointestinal pathologists who were blinded to all clinical, endoscopic, and biomarker data. Inflammatory activity was graded using the Geboes scoring system [28].

Histologic grading was performed according to the original Geboes score classification as described by Karel Geboes et al. Histologic healing was predefined as a Geboes score strictly below 2.0, whereas histologic activity was defined as a Geboes score ≥ 2.0, corresponding to the presence of neutrophilic infiltration and active microscopic inflammation. This threshold was selected a priori based on its frequent use in studies evaluating histologic remission in inflammatory bowel disease. Because neutrophilic infiltration represents a clinically relevant marker of active microscopic inflammation, an additional exploratory sensitivity analysis was performed using the stricter threshold corresponding to Geboes grade ≥ 2B.1.

For analytical consistency, the maximum Geboes score recorded for each patient was used to represent overall histologic disease activity. This approach was selected to ensure that focal areas of inflammation were not underestimated and to enable consistent integration of histologic findings into downstream phenotype classification and patient-level stratification analyses.

### 2.4. Endoscopic–Histologic Phenotype Classification

To facilitate clinically meaningful stratification, patients were categorized according to the combined status of endoscopic and histologic disease activity. This approach was used to delineate biologically distinct states of disease control, rather than to perform descriptive subgroup comparisons alone.

Three phenotypic categories were defined. Patients were considered to be in concordant remission when both endoscopic and histologic assessments indicated inactive disease. Discordant disease was defined as the presence of histologic inflammation despite endoscopic remission, reflecting residual microscopic activity. Concordant active disease was defined by simultaneous evidence of endoscopic and histologic inflammation.

A small subset of patients (*n* = 2) demonstrated endoscopic activity without corresponding histologic inflammation. Given the limited number of such cases and the lack of analytical stability, these patients were not included in phenotype-based comparative analyses but were retained for descriptive reporting. Given the observed imbalance in disease subtype distribution across endoscopic–histologic phenotypes and the limited number of Crohn’s disease cases with discordant disease, the primary analytical framework was prospectively restricted to patients with ulcerative colitis (UC) in endoscopic remission. Exploratory pooled UC/CD analyses were additionally performed as sensitivity analyses.

The primary analytical framework focused on patients in endoscopic remission, within whom the distinction between concordant remission and discordant disease was considered clinically relevant. This restriction allowed for targeted evaluation of residual microscopic inflammation in a population otherwise classified as being in remission.

Rather than serving solely as descriptive categories, these phenotypes were subsequently used as reference states for the development of a cytokine-based stratification model, enabling the translation of biological differences into patient-level risk classification.

### 2.5. Blood Sampling and Cytokine Quantification

Peripheral venous blood samples were collected immediately prior to colonoscopy and before any procedural intervention, in order to ensure that cytokine measurements reflected baseline systemic inflammatory status.

Following collection, samples were processed within two hours. Serum was separated by centrifugation and stored at −80 °C under standardized conditions until analysis. To minimize pre-analytical variability, all samples were handled using a uniform protocol across the study population, including consistent processing times, storage conditions, and freeze–thaw exposure.

Cytokine measurements were performed in batch during a defined analytical period to reduce inter-assay variability. The median storage duration was approximately three months (range: 1–6 months), storage duration was comparable across study groups, and all samples underwent a single freeze–thaw cycle prior to analysis. Quantification of interleukin-10 (IL-10) and interleukin-23 (IL-23) was performed in duplicate, with mean values used for downstream analyses. Values below the assay lower limit of detection (LOD) were handled according to a predefined analytical strategy. For primary analyses, assay-reported values were retained for modeling, whereas values below the LOD were imputed as LOD/2 for descriptive analyses. Additional sensitivity analyses were performed using raw assay-reported extrapolated IL-10 values without LOD-based substitution to evaluate the robustness of biomarker performance estimates.

Assay performance was monitored using standard calibration curves and internal quality controls, in accordance with the manufacturer’s instructions. The reported intra-assay and inter-assay coefficients of variation were <10% for both cytokines. All laboratory procedures were conducted under standardized conditions by experienced personnel who were blinded to clinical, endoscopic, and histologic data. The selection of IL-10 and IL-23 was hypothesis-driven, aiming to capture the balance between regulatory (anti-inflammatory) and pro-inflammatory immune pathways within a simplified and clinically interpretable framework.

### 2.6. Interleukin-10 (IL-10) Measurement and Data Handling

Serum IL-10 concentrations were determined using a commercially available Quantikine ELISA kit (R&D Systems, Minneapolis, MN, USA; Cat. No. D1000B) in accordance with the manufacturer’s protocol. All samples and standards were analyzed in duplicate, and mean values were used for subsequent analyses. Absorbance was measured at 450 nm with wavelength correction using a microplate reader (BioTek Instruments, Winooski, VT, USA).

The lower limit of detection (LOD) for the assay was 3.9 pg/mL. To preserve the continuous nature of the biomarker signal, assay-derived IL-10 values were retained for analytical modeling without truncation.

For descriptive and tabular purposes, values below the LOD were imputed as half the detection threshold (LOD/2) to reduce bias associated with zero-inflated distributions and to maintain comparability across groups.

In addition to continuous modeling, IL-10 was further evaluated using a clinically interpretable threshold (≥3.9 pg/mL vs. <3.9 pg/mL), derived from receiver operating characteristic (ROC) analysis, to support downstream risk stratification. However, given the observed distribution of IL-10 values, with a substantial proportion clustering at or below the lower limit of detection, IL-10 effectively behaved as a threshold-driven biomarker in this cohort. Therefore, its discriminatory performance should be interpreted as primarily reflecting the distinction between detectable and non-detectable levels, rather than a fully continuous biological gradient. To evaluate the robustness of this approach, an additional sensitivity analysis was performed using raw assay-reported extrapolated IL-10 values without LOD/2 substitution.

### 2.7. Interleukin-23 (IL-23) Measurement and Analytical Approach

Serum IL-23 concentrations were measured using a Quantikine ELISA kit (R&D Systems, Minneapolis, MN, USA; Cat. No. D2300C) in accordance with the manufacturer’s protocol. All samples were analyzed in duplicate, and mean values were used for subsequent analyses.

The lower limit of detection (LOD) for IL-23 was 16.3 pg/mL. Although the assay provides extrapolated quantitative values below this threshold, the distribution of IL-23 concentrations in the study population was characterized by a high proportion of low or near-undetectable values.

To account for this distributional pattern, two complementary analytical strategies were applied. For descriptive purposes, values below the LOD were imputed as half the detection threshold (LOD/2). However, given the limited variability of IL-23 as a continuous variable, primary analyses incorporated IL-23 as a categorical marker (detectable ≥ 16.3 pg/mL vs. non-detectable), enabling more robust and clinically interpretable stratification. Receiver operating characteristic (ROC)-derived analyses demonstrated limited discriminatory performance of IL-23 as an isolated biomarker; therefore, its primary analytical role was exploratory refinement of cytokine-based subgroup classification rather than standalone prediction.

The combined evaluation of IL-10 and IL-23 was designed to reflect the balance between regulatory and pro-inflammatory immune signaling, and to support the development of a simplified cytokine-based framework for patient-level risk classification. Although additional cytokines, such as IL-17 and IL-33, are known to play important roles in IBD pathophysiology, the present study focused on a limited number of biomarkers to preserve model simplicity and clinical interpretability.

### 2.8. Handling of Detection Limits and Biomarker Representation

Cytokine measurements were incorporated into the analytical framework using approaches tailored to their distributional characteristics and intended clinical application. To preserve the underlying variability of the data, assay-reported values were retained for continuous analyses, including measurements below the nominal limits of detection (LOD).

Manufacturer-defined lower detection limits were 3.9 pg/mL for IL-10 and 16.3 pg/mL for IL-23. Nevertheless, calibration curve fitting allowed estimation of concentrations at the lower end of the measurable spectrum.

For descriptive purposes, values below the LOD were imputed as half the respective detection threshold (LOD/2), enabling consistent summary statistics and avoiding distortion due to zero-inflated distributions. These imputations were not applied in primary modeling analyses. To evaluate the potential influence of LOD-based handling on biomarker performance, additional sensitivity analyses were performed using raw assay-reported extrapolated IL-10 values without LOD/2 substitution.

Given the differing distributional profiles of the two cytokines, additional representations were considered to enhance clinical interpretability. Given the observed distribution of IL-10 values, with substantial clustering at or below the lower limit of detection, IL-10 effectively behaved as a threshold-based biomarker in this cohort. Therefore, its performance should be interpreted as reflecting detectability rather than a fully continuous biological gradient. In contrast, IL-23 was additionally evaluated as a categorical variable (detectable vs. non-detectable), due to the high proportion of low or near-undetectable values.

This dual representation allowed integration of both biomarkers into a unified analytical framework, supporting the development of clinically interpretable thresholds and subsequent patient-level risk stratification. Importantly, primary modeling analyses were based on assay-reported values rather than imputed data, and the use of LOD/2 substitution was restricted to descriptive summaries.

### 2.9. Routine Laboratory Parameters and Clinical Context

Standard laboratory measurements, including C-reactive protein (CRP), hemoglobin, and white blood cell (WBC) count, were obtained as part of routine clinical assessment using automated assays in an accredited hospital laboratory.

These parameters were incorporated to provide contextual information on systemic inflammatory burden and overall hematologic status. In particular, CRP was evaluated as a conventional inflammatory marker to allow comparison between cytokine-based and standard laboratory approaches in the detection of residual disease activity. CRP was included as a routinely available systemic biomarker; however, it is recognized that it lacks specificity for intestinal inflammation and may be influenced by extra-intestinal factors.

Hemoglobin and WBC counts were included for descriptive characterization of the study population and to support the clinical interpretation of endoscopic–histologic phenotypes.

### 2.10. Study Outcomes

The primary outcome of the study was the presence of histologic activity among patients with endoscopic remission, defined as a Geboes score ≥ 2.0. This outcome was selected to capture residual microscopic inflammation in a clinically relevant population otherwise considered to be in remission. An additional exploratory sensitivity analysis was performed using the stricter histologic activity threshold corresponding to Geboes grade ≥ 2B.1.

The primary objective was not limited to group-level comparisons but focused on the development and evaluation of a cytokine-based framework for identifying patients at increased risk of persistent histologic inflammation. Accordingly, the study aimed to assess the discriminatory performance of circulating cytokines and to translate these signals into clinically interpretable thresholds.

Secondary outcomes included the evaluation of individual biomarker performance using receiver operating characteristic (ROC) curve analysis, as well as the identification of optimal cut-off values for interleukin-10 (IL-10), interleukin-23 (IL-23), and C-reactive protein (CRP).

An additional predefined objective was the construction of a combined cytokine-based model to enable patient-level risk stratification. Patients were categorized into low-, intermediate-, and high-risk groups based on predefined cytokine thresholds, and the association between risk category and histologic activity was evaluated.

Finally, multivariable modeling was performed to assess the independent contribution of each biomarker to the prediction of histologic activity, using penalized logistic regression to account for small sample size and potential separation.

### 2.11. Statistical Analysis

Continuous variables are presented as median with interquartile range (IQR), and categorical variables as counts and percentages. Due to the non-normal distribution of several variables, nonparametric methods were applied throughout. All statistical tests were two-sided, with a significance threshold set at *p* < 0.05.

Baseline comparisons across endoscopic–histologic phenotypes were performed using the Kruskal–Wallis test for continuous variables and the chi-square or Fisher’s exact test for categorical variables, as appropriate. Specifically, the Kruskal–Wallis test was used for comparisons across the three phenotype groups, while pairwise comparisons within the endoscopic remission subgroup were performed using the Mann–Whitney U test. For analyses restricted to the endoscopic remission cohort, comparisons between concordant remission and discordant disease were conducted using the Mann–Whitney U test and Fisher’s exact test. Additional exploratory analyses stratified by sex were performed using the Mann–Whitney U test for continuous variables and Fisher’s exact test for categorical variables. For exploratory treatment-stratified analyses, advanced biologic therapy and combination therapy were categorized as biologic therapy, whereas 5-aminosalicylic acid (5-ASA), conventional non-biologic therapy, and no treatment were categorized as non-biologic therapy.

To evaluate the diagnostic performance of biomarkers for detecting histologic activity in patients with endoscopic remission, receiver operating characteristic (ROC) curve analysis was performed. Optimal cut-off values were determined using the Youden index, and corresponding sensitivity and specificity estimates were calculated. ROC analyses were performed using predicted probabilities derived from each biomarker or model, and AUC values were used to quantify overall discrimination. As a sensitivity analysis, ROC analysis for IL-10 was additionally repeated using raw assay-reported extrapolated values without LOD/2 substitution in order to evaluate the potential impact of left-censored data handling on biomarker performance.

Based on these predefined thresholds, cytokines were incorporated into a clinically interpretable framework using binary representations (IL-10 < 3.9 pg/mL; IL-23 ≥ 16.3 pg/mL). These variables were subsequently used to construct a combined cytokine-based model for patient-level risk stratification. Patients were categorized into low-, intermediate-, and high-risk groups, and the association between risk category and histologic activity was assessed using the chi-square test. The chi-square test was applied to evaluate overall differences across risk categories, with Fisher’s exact test used when expected cell counts were small.

Multivariable analysis was performed to evaluate the independent contribution of cytokine-based predictors using Firth penalized logistic regression, in order to account for small sample size and quasi-complete separation. Odds ratios (ORs) with 95% confidence intervals (CIs) were reported. Firth penalization was specifically applied to reduce bias in parameter estimation under conditions of limited events per variable. Internal validation of model performance was performed using bootstrap resampling (1000 iterations), with estimation of the area under the curve (AUC) and corresponding confidence intervals to assess the stability of discrimination and potential optimism in model performance. Model comparison between nested models was performed using the likelihood-ratio test to evaluate the incremental value of IL-23 beyond IL-10. An additional predefined sensitivity analysis was performed using the stricter histologic activity definition corresponding to Geboes ≥ 2B.1. Because no patient reclassification occurred under this stricter threshold, all primary analyses yielded identical subgroup allocation and model performance estimates.

Given the presence of left-censored cytokine values, Tobit and interval-censored regression approaches were considered. However, because the primary outcome was binary histologic activity and IL-10 demonstrated threshold-driven behavior related to assay detectability, the primary analytical framework prioritized clinically interpretable binary biomarker representations. Given the exploratory nature of the study and the limited sample size, no formal adjustment for multiple comparisons was performed; results should therefore be interpreted cautiously.

All analyses were designed with an emphasis on clinical interpretability and were considered exploratory and hypothesis-generating. Statistical analyses were performed using R software (version 4.3.1; R Foundation for Statistical Computing, Vienna, Austria).

## 3. Results

### 3.1. Study Population and Phenotype Classification

A total of 59 patients with inflammatory bowel disease (IBD) and complete clinical, endoscopic, histologic, and biomarker data were included in the overall study cohort. The population comprised patients with ulcerative colitis (UC) and Crohn’s disease (CD) undergoing colonoscopy as part of routine clinical care.

Based on the combined assessment of endoscopic and histologic findings, patients were categorized into three predefined endoscopic–histologic phenotypes. Concordant remission, defined as the coexistence of endoscopic remission and histologic healing, was observed in 22 patients. Discordant disease, defined as persistent histologic activity despite endoscopic remission, was identified in 14 patients. Concordant active disease, characterized by the presence of both endoscopic and histologic activity, was observed in 21 patients.

A small number of patients (*n* = 2) exhibited endoscopic activity in the absence of histologic inflammation. Due to the limited number of such cases, these patients were excluded from phenotype-based comparative analyses but were retained for descriptive completeness.

Given the observed imbalance in disease subtype distribution across phenotypes, with ulcerative colitis more frequently represented in the discordant subgroup, and considering the limited number of Crohn’s disease cases with endoscopic–histologic discordance, the primary analytical framework was restricted to patients with ulcerative colitis (UC). Consequently, subsequent analyses focused on patients with UC in endoscopic remission, comparing concordant remission and discordant disease in order to evaluate biomarkers associated with residual histologic activity in a clinically relevant remission population. Exploratory pooled UC/CD analyses were retained as supplementary sensitivity analyses.

Baseline characteristics of the study population across the three endoscopic–histologic phenotypes are presented in Table 1. Current treatment exposure was additionally summarized across UC phenotype groups to explore the potential influence of biologic therapy on circulating cytokine patterns. No significant differences in treatment distribution were observed between phenotype groups (χ^2^ *p* = 0.699). These data are presented in Supplementary Table S1.

Baseline clinical, laboratory, and biomarker characteristics stratified by sex are presented in Table 2. Female patients demonstrated lower hemoglobin levels compared with male patients (12.7 vs. 14.0 g/dL, *p* = 0.001), whereas no statistically significant sex-specific differences were observed for age, disease duration, CRP, IL-10, IL-23, or WBC counts. A modest non-significant trend toward higher IL-23 levels was observed among female patients. Given the exploratory nature of these analyses and the limited sample size, sex-specific findings should be interpreted cautiously.

### 3.2. Biomarker Performance for Detection of Histologic Activity

To evaluate the ability of circulating biomarkers to detect residual histologic activity, receiver operating characteristic (ROC) curve analysis was performed in patients with ulcerative colitis (UC) in endoscopic remission.

Among the evaluated biomarkers, interleukin-10 (IL-10) demonstrated the strongest discriminatory performance for identifying histologic activity. When analyzed using LOD-adjusted values, IL-10 achieved an area under the curve (AUC) of 0.850 (95% CI 0.704–0.996). Lower or non-detectable IL-10 levels were associated with the presence of residual microscopic inflammation, and ROC analysis identified a threshold corresponding to the assay lower limit of detection (<3.9 pg/mL), effectively distinguishing detectable from non-detectable IL-10 levels. At this threshold, IL-10 demonstrated sensitivity of 84.6% and specificity of 77.8%, supporting its role as a sensitive screening biomarker.

Notably, the identified threshold (<3.9 pg/mL) corresponds to the assay lower limit of detection and therefore reflects the distinction between detectable and non-detectable IL-10 levels rather than a purely biologically derived cut-off. To evaluate the potential influence of LOD-based imputation on biomarker performance, a sensitivity ROC analysis was additionally performed using raw assay-reported extrapolated IL-10 values without LOD/2 substitution. In this analysis, IL-10 retained strong discriminatory performance (AUC 0.906, 95% CI 0.789–1.000), with sensitivity of 92.3% and specificity of 77.8%, supporting the robustness of the observed association.

In contrast, interleukin-23 (IL-23), evaluated as a categorical variable based on detectability (≥16.3 pg/mL), demonstrated limited discriminatory performance overall (AUC 0.615, 95% CI 0.402–0.829), characterized by low sensitivity (23.1%) but high specificity (94.4%). These findings suggest that detectable IL-23 may identify a small subgroup of patients with increased likelihood of histologic activity, although its standalone predictive utility remained limited in this cohort.

C-reactive protein (CRP) demonstrated poor discriminatory performance (AUC 0.581, 95% CI 0.364–0.798), with limited sensitivity and specificity for distinguishing histologic activity states within the endoscopic remission population. Detailed exploratory ROC analyses for CRP are provided in Supplementary Table S2.

Given the exploratory nature of the study and the limited sample size, all biomarker performance estimates should be interpreted cautiously. The diagnostic characteristics of IL-10 and IL-23, including ROC-derived cut-off values, sensitivity, specificity, and AUC estimates, are summarized in Table 3.

### 3.3. Cytokine-Based Risk Stratification of Histologic Activity

Building upon the predefined cytokine thresholds, a combined IL-10 and IL-23 framework call was developed to enable patient-level risk stratification in patients with ulcerative colitis (UC) in endoscopic remission.

Patients were categorized into four predefined risk subgroups according to the combined status of IL-10 and IL-23. The low-risk subgroup was defined by preserved IL-10 levels (≥3.9 pg/mL) and non-detectable IL-23 (<16.3 pg/mL). The high-risk subgroup was defined by reduced IL-10 levels (<3.9 pg/mL) in combination with detectable IL-23 (≥16.3 pg/mL). To further explore heterogeneity within the intermediate-risk category, patients were subdivided into Intermediate A (IL-10 ≥ 3.9 pg/mL and IL-23 ≥ 16.3 pg/mL) and Intermediate B (IL-10 < 3.9 pg/mL and IL-23 < 16.3 pg/mL).

This stratification approach identified clinically distinct subgroups with progressively increasing observed proportions of histologic activity. Histologic inflammation was observed in only 1 of 14 patients (7.1%) in the low-risk subgroup, compared with 1 of 2 patients (50.0%) in Intermediate A and 9 of 12 patients (75.0%) in Intermediate B. Notably, all patients in the high-risk subgroup (3/3, 100%) demonstrated persistent histologic activity.

The association between cytokine-defined risk categories and histologic activity was statistically significant (*p* < 0.001), supporting the ability of this framework to distinguish clinically meaningful differences within the UC endoscopic remission cohort. Importantly, all risk-stratification analyses presented in the primary framework were restricted to patients with ulcerative colitis.

These findings suggest that the integration of regulatory (IL-10) and pro-inflammatory (IL-23) immune signals may support exploratory patient-level stratification according to the observed likelihood of residual microscopic inflammation. Most histologic activity events occurred among patients with reduced IL-10 levels, whereas detectable IL-23 appeared to identify a small subgroup with particularly high observed risk, although this estimate was based on very limited patient numbers and should therefore be interpreted cautiously. The complete distribution of patients across cytokine-defined risk categories is presented in Table 4.

Exploratory pooled UC/CD analyses were additionally performed as sensitivity analyses and are provided in Supplementary Table S3. These findings were directionally consistent with the primary UC-restricted analysis; however, because the pooled cohort was predominantly composed of patients with ulcerative colitis and included a limited number of Crohn’s disease cases with discordant endoscopic–histologic activity, CD-specific inferences cannot be reliably supported.

**Figure 1:**
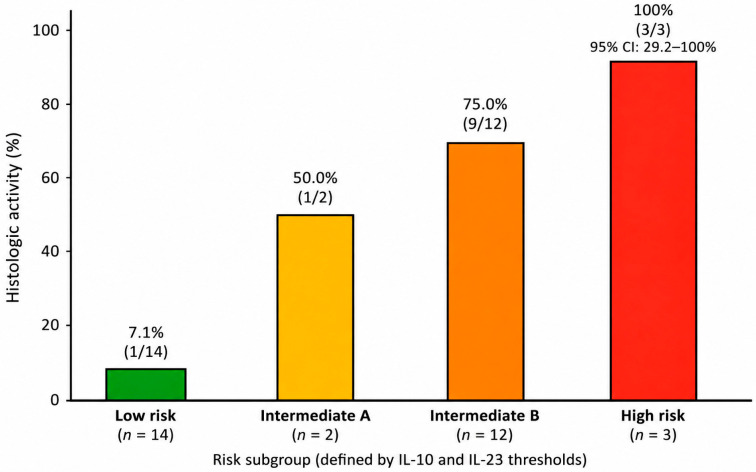
Cytokine-based risk stratification of histologic activity in patients with ulcerative colitis (UC) in endoscopic remission. Patients were classified into low-, intermediate-, and high-risk groups according to predefined IL-10 and IL-23 thresholds. The green bar represents the low-risk group, the yellow bar represents Intermediate A, the orange bar represents Intermediate B, and the red bar represents the high-risk group. The observed proportion of patients with histologic activity increased progressively across risk categories, from 7.1% in the low-risk group to 50.0% in Intermediate A, 75.0% in Intermediate B, and 3/3 patients (100%; 95% CI 29.2–100%) in the high-risk group. Overall, this pattern supports the clinical interpretability of the proposed stratification framework, although the findings should be considered exploratory. The high-risk subgroup consisted of only three patients, and the corresponding estimate should therefore be interpreted cautiously.

A graphical representation of cytokine-based risk stratification is provided to enhance interpretability (Figure 1).

**Figure 2:**
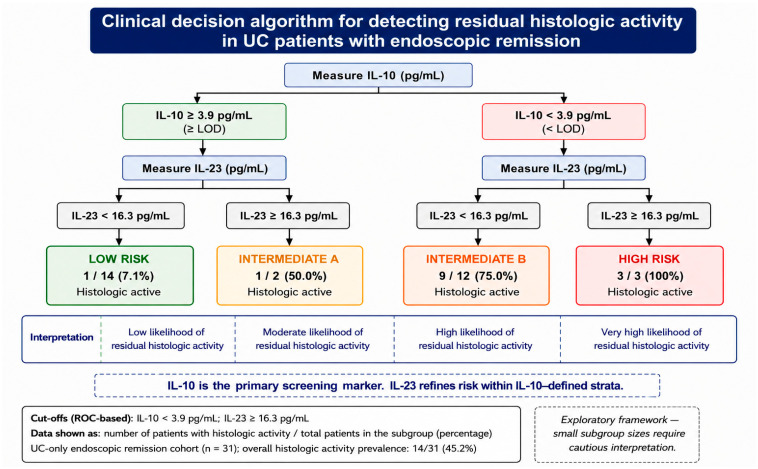
Exploratory cytokine-based clinical decision framework for detecting residual histologic activity in patients with ulcerative colitis (UC) in endoscopic remission. A two-step cytokine-based approach using IL-10 and IL-23 may enable patient-level risk stratification. IL-10 serves as the primary screening marker, while IL-23 further refines classification by identifying patients with increased likelihood of residual histologic activity within IL-10–defined strata. The combined model categorizes patients into low-risk, Intermediate A, Intermediate B, and high-risk groups, potentially informing individualized consideration of biopsy and therapeutic assessment. The displayed proportions represent observed frequencies within this cohort and should not be interpreted as population-level risk estimates. Given the small subgroup sizes, these findings should be considered exploratory and interpreted with caution. The high-risk subgroup included only three patients; therefore, the observed proportion should be interpreted cautiously.

Based on these findings, a simplified cytokine-based clinical decision algorithm was developed to support patient-level risk stratification (Figure 2).

### 3.4. Multivariable Analysis and Model Performance

To further evaluate the independent contribution of cytokine-based predictors, multivariable analysis was performed in patients with ulcerative colitis (UC) in endoscopic remission using Firth penalized logistic regression.

In this model, reduced IL-10 levels (<3.9 pg/mL) were independently associated with the presence of histologic activity (OR 15.55, 95% CI 2.63–172.70; *p* = 0.0016), although the estimate was characterized by a wide confidence interval reflecting statistical uncertainty related to the limited sample size. In contrast, detectable IL-23 levels (≥16.3 pg/mL) were not independently associated with histologic activity (OR 4.05, 95% CI 0.30–584.58; *p* = 0.325), likely reflecting the very small number of patients with detectable IL-23 and the resulting instability of subgroup-specific estimates.

Receiver operating characteristic (ROC) analysis confirmed the strong discriminatory performance of IL-10, both when analyzed using LOD-adjusted values (AUC 0.850) and raw assay-reported extrapolated values (AUC 0.906). In contrast, IL-23 and CRP demonstrated limited predictive performance, with AUC values of 0.615 and 0.581, respectively. The combined IL-10 + IL-23 model achieved an AUC of 0.855, indicating similar overall discrimination compared with IL-10 alone. These findings suggest that IL-10 captures most of the predictive signal for residual histologic activity in this cohort.

Given that model development and evaluation were performed within the same dataset, the reported performance estimates may be subject to optimism bias. Internal validation using bootstrap resampling (1000 iterations) demonstrated consistent discrimination, although confidence intervals remained wide, reflecting the exploratory nature of the analysis and the limited cohort size.

Despite the absence of substantial improvement in overall discrimination, the combined IL-10 + IL-23 framework enhanced clinical interpretability through cytokine-based risk stratification. This suggests that IL-23 may contribute primarily to refinement of patient classification rather than to improvement of predictive accuracy.

The results of multivariable analysis and ROC curve evaluation are presented in Table 5 and Table 6. To formally evaluate the incremental contribution of IL-23 beyond IL-10, nested model comparison was performed using a likelihood-ratio test. The addition of IL-23 did not significantly improve model fit (LR = 2.35, *p* = 0.13; Table 7).

**Table 5:** Firth logistic regression for histologic activity in ulcerative colitis.

Variable	OR	95% CI	*p*-Value
IL-10 < 3.9 pg/mL	15.55	2.63–172.70	0.0016
IL-23 ≥ 16.3 pg/mL	4.05	0.30–584.58	0.325

**Figure 3:**
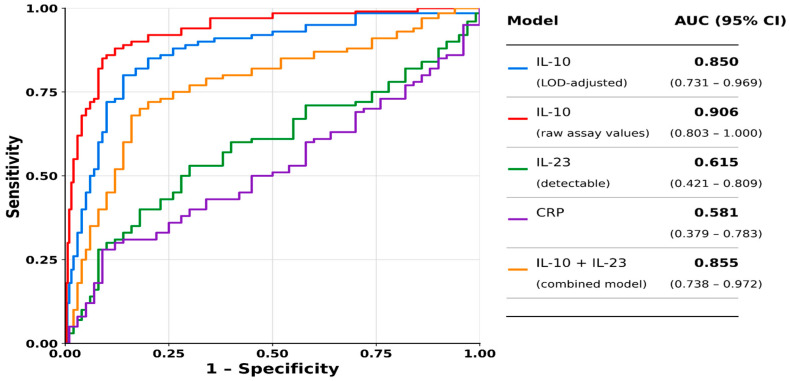
Receiver operating characteristic (ROC) curves for the detection of histologic activity in patients with ulcerative colitis (UC) in endoscopic remission. IL-10 demonstrated the strongest discriminatory performance, both when analyzed as LOD-adjusted values (AUC 0.850, 95% CI 0.731–0.969) and as raw assay values (AUC 0.906, 95% CI 0.803–1.000). In contrast, IL-23 (detectable; AUC 0.615, 95% CI 0.421–0.809) and C-reactive protein (CRP; AUC 0.581, 95% CI 0.379–0.783) showed limited predictive performance. The combined IL-10 + IL-23 model achieved an AUC of 0.855 (95% CI 0.738–0.972), which did not substantially improve discrimination compared with IL-10 alone, suggesting that IL-10 captures most of the predictive signal. Interpretation of IL-10 performance should consider its threshold behavior in relation to the assay detection limit (LOD).

Model: Firth penalized logistic regression. Outcome: histologic activity among patients with ulcerative colitis in endoscopic remission. Footnote: Firth penalized logistic regression was performed to evaluate the association between cytokine-based predictors and histologic activity in patients with ulcerative colitis (UC) in endoscopic remission, in order to account for small sample size and quasi-complete separation. Variables were entered as binary predictors based on predefined ROC-derived cut-off values (IL-10 < 3.9 pg/mL; IL-23 ≥ 16.3 pg/mL). Odds ratios (ORs) with 95% confidence intervals (CIs) are reported. Reduced IL-10 levels were independently associated with histologic activity, whereas detectable IL-23 did not demonstrate an independent association, likely reflecting the limited number of patients with detectable IL-23 and the resulting statistical instability. Graphical representations of ROC analyses are provided in Figure 3.

**Table 6:** ROC curve analysis for detection of histologic activity in UC endoscopic remission.

Model	AUC	Interpretation
IL-10 (LOD-adjusted)	0.850	Strong discrimination
IL-10 (raw assay-reported values)	0.906	Strong discrimination
IL-23 (detectable)	0.615	Poor–moderate
CRP	0.581	Poor
IL-10 + IL-23 (combined)	0.855	Strong discrimination

Footnote: Receiver operating characteristic (ROC) curve analysis was performed in patients with ulcerative colitis (UC) in endoscopic remission to evaluate the discriminatory performance of cytokine-based and conventional inflammatory biomarkers for detecting histologic activity. The combined IL-10 + IL-23 model was derived from logistic regression including binary cytokine variables based on predefined ROC-derived cut-off values, and predicted probabilities were used to calculate the AUC. IL-10 demonstrated the strongest overall discriminatory performance, whereas IL-23 and CRP showed limited predictive value. The addition of IL-23 to IL-10 did not substantially improve overall discrimination, suggesting that IL-10 captures most of the predictive signal in this cohort. Internal validation was performed using bootstrap resampling (1000 iterations).

**Table 7:** Incremental value of IL-23: model comparison in ulcerative colitis.

Model	AUC	Log-Likelihood
IL-10 model	0.823	−13.90
IL-10 + IL-23 model	0.855	−12.73

Likelihood-ratio test: LR = 2.35, *p* = 0.13. Footnote: Nested model comparison was performed using a likelihood-ratio (LR) test to evaluate the incremental contribution of IL-23 beyond IL-10 for the detection of histologic activity in patients with ulcerative colitis (UC) in endoscopic remission. The IL-10 + IL-23 combined model demonstrated a numerically higher AUC compared with the IL-10-only model; however, the improvement in model fit was not statistically significant (LR = 2.35, *p* = 0.13). These findings suggest that IL-10 captures most of the predictive signal in this cohort, whereas IL-23 may contribute primarily to refinement of patient-level risk stratification rather than substantial improvement in overall discrimination. AUC values were derived from receiver operating characteristic (ROC) analysis using predicted probabilities generated from each model.

To evaluate whether the proposed framework was dependent on the selected definition of histologic activity, an additional sensitivity analysis was performed using the stricter threshold corresponding to neutrophilic inflammation (Geboes score ≥ 2B.1). No patient reclassification was observed compared with the predefined primary threshold of Geboes ≥ 2.0, as all patients classified with histologic activity under the primary definition already demonstrated neutrophilic inflammation. Consequently, ROC analyses, multivariable model performance, and cytokine-based risk stratification results remained unchanged. The results of this sensitivity analysis are summarized in Table 8.

## 4. Discussion

The present study addresses a clinically relevant but insufficiently operationalized problem in inflammatory bowel disease (IBD): the identification of residual microscopic inflammation in patients who have already achieved endoscopic remission. Rather than further characterizing cytokine differences across disease states, our approach focuses on translating systemic immune signals into a clinically applicable framework for individualized risk assessment [29,30,31,32].

Within a population conventionally classified as being in remission, we observed substantial biological heterogeneity, with a considerable proportion of patients exhibiting ongoing histologic activity. This finding reinforces the concept that endoscopic healing represents a necessary but not sufficient condition for complete disease control [33,34,35]. More importantly, it highlights the need for tools capable of distinguishing between biologically distinct states that are not apparent through routine clinical assessment. Importantly, the primary analytical framework of the present study was restricted to patients with ulcerative colitis (UC), given the observed imbalance in disease subtype distribution across phenotypes and the limited number of Crohn’s disease cases with endoscopic–histologic discordance. Therefore, the current findings should primarily be interpreted within the context of UC.

In this context, the integration of interleukin-10 (IL-10) and interleukin-23 (IL-23) enabled the development of a simplified cytokine-based stratification model that categorizes patients into low-, intermediate-, and high-risk groups. This framework does not aim to replace existing biomarkers or diagnostic modalities, but rather to provide an additional layer of biological resolution within clinically defined remission states [36,37,38]. Notably, the observed proportion of histologic activity increased progressively across cytokine-defined subgroups; however, estimates for the smaller subgroups, particularly the high-risk subgroup, should be interpreted cautiously because of the limited number of included patients.

Importantly, established biomarkers such as fecal calprotectin remain the cornerstone of non-invasive disease monitoring in IBD, given their strong correlation with mucosal inflammation and extensive clinical validation. Therefore, the proposed cytokine-based framework should be interpreted as complementary and exploratory, rather than as an alternative to current standard-of-care biomarkers.

The absence of fecal calprotectin in the present study represents a key limitation, as it precludes direct comparison with the established non-invasive standard for assessing mucosal inflammation. Consequently, the incremental clinical value of the proposed cytokine-based stratification framework relative to current monitoring strategies cannot be determined. Therefore, the present findings should be interpreted as exploratory and complementary, rather than as evidence supporting added clinical utility over existing biomarkers.

A central observation of this study is that the value of combining IL-10 and IL-23 lies not in improving overall discrimination, but in enhancing clinical interpretability [39]. Indeed, the combined IL-10 + IL-23 model did not demonstrate a meaningful improvement in discriminatory performance compared with IL-10 alone, as reflected by similar AUC values. This finding suggests that IL-10 captures the majority of the predictive signal. While IL-10 demonstrated strong discriminatory performance, its behavior in this cohort was strongly influenced by detectability at the assay lower limit of detection. Sensitivity analysis using raw assay-reported extrapolated IL-10 values without LOD/2 substitution demonstrated similarly strong discriminatory performance, supporting the robustness of the observed association despite the threshold-driven behavior of IL-10 in this cohort. The addition of IL-23 allowed the identification of patients at the extremes of risk, including a subgroup with a high observed probability of persistent histologic inflammation, although this estimate is based on a very small number of patients and should be interpreted with caution.

Therefore, the contribution of IL-23 should be considered primarily as a refinement tool for risk stratification rather than a driver of predictive accuracy. Although formal reclassification metrics (such as net reclassification improvement and integrated discrimination improvement) were considered, their interpretation is limited by the very small number of patients with detectable IL-23, and therefore, these analyses were not considered robust in the present dataset. This distinction is particularly relevant in the context of personalized medicine, where the objective is not solely accurate prediction, but actionable classification that can guide clinical decisions at the individual level [40].

Importantly, the IL-10 threshold identified in this study corresponds to the assay lower limit of detection and should therefore be interpreted as a detectability-based threshold rather than a purely biologically derived cut-off. This limitation supports interpreting IL-10 as a threshold-driven biomarker in the present cohort, rather than as a fully continuous marker of residual histologic activity.

From a mechanistic perspective, the observed patterns can be interpreted as reflecting the balance between regulatory and pro-inflammatory immune signaling. However, the primary contribution of this study is not the biological interpretation per se, but the translation of these signals into an exploratory yet clinically interpretable framework [41,42,43]. By reducing complex immunologic information into a small number of interpretable variables, this approach facilitates integration into routine clinical workflows.

The limited performance of C-reactive protein (CRP) within the endoscopic remission cohort further emphasizes the need for alternative strategies. Conventional systemic markers appear insufficient to capture low-grade or compartmentalized inflammation, supporting the concept that immune-specific biomarkers may offer incremental value in selected clinical contexts.

Importantly, the proposed framework may align with a stepwise clinical approach. Patients classified as low risk may potentially require less intensive histologic reassessment, although this hypothesis requires prospective validation. Intermediate-risk patients represent a heterogeneous group in whom additional clinical or biomarker data may be necessary. Such a model may reflect a shift from uniform monitoring strategies toward risk-adapted decision-making.

Several limitations should be considered when interpreting these findings. First, the relatively small sample size and single-center design may limit generalizability and contribute to statistical uncertainty, particularly within subgroup analyses. Although appropriate statistical techniques, including penalized regression, were applied to mitigate small-sample bias, the resulting estimates remain imprecise, as reflected by wide confidence intervals. Although exploratory sex-stratified analyses were performed, the limited sample size precluded robust sex-specific inference. Furthermore, the small number of events and the very limited size of certain subgroups, particularly the high-risk category, restrict the stability of subgroup-specific estimates and require cautious interpretation. Importantly, sensitivity analysis using the stricter Geboes ≥ 2B.1 definition yielded identical patient classification and model performance estimates within the present cohort, supporting the robustness of the findings across histologic activity thresholds.

Second, the cross-sectional design restricts the analysis to a single time-point assessment and does not allow evaluation of temporal dynamics. As such, the ability of the proposed cytokine-based framework to predict longitudinal outcomes, including relapse, treatment response, or disease progression, remains uncertain and requires prospective validation.

Third, although exploratory pooled UC/CD analyses were performed as sensitivity analyses, the primary analytical framework was restricted to patients with ulcerative colitis because of the observed imbalance in disease subtype distribution across phenotypes and the limited number of Crohn’s disease cases with endoscopic–histologic discordance. Consequently, the present findings should primarily be interpreted within the context of UC, and disease-specific validation in adequately powered Crohn’s disease cohorts remains necessary.

In addition, treatment exposure represents a potential source of confounding, given the known immunomodulatory effects of biologic and immunosuppressive therapies on cytokine profiles. Although key sources of bias were minimized through study design and predefined exclusion criteria, residual confounding cannot be fully excluded.

The absence of fecal calprotectin, a well-established biomarker of intestinal inflammation, represents a fundamental limitation, as it prevents direct assessment of the incremental clinical value of cytokine-based stratification within existing monitoring frameworks. CRP was included as a routinely available systemic marker for comparison; however, it lacks specificity for intestinal inflammation and may be influenced by extra-intestinal factors. Importantly, it was not intended as a primary comparator but rather as a contextual reference reflecting standard clinical practice. Future studies should evaluate combined biomarker approaches to better define clinical utility.

In addition, the selection of cytokines was limited to IL-10 and IL-23, based on a hypothesis-driven approach aimed at capturing the balance between regulatory and pro-inflammatory immune pathways within a simplified and clinically interpretable framework. Although other cytokines, such as IL-17 and IL-33, are known to play important roles in IBD pathophysiology, their evaluation was beyond the scope of the present study and may provide additional biological and predictive information in future research.

Furthermore, systemic cytokine measurements may not fully reflect mucosal immune activity, as local immune processes within the intestinal microenvironment may differ from circulating levels. Accordingly, these biomarkers should be interpreted as indirect indicators of underlying inflammation rather than direct surrogates.

A further limitation is that model development and evaluation were performed within the same dataset; therefore, the reported performance estimates may be subject to optimism bias. Although internal validation using bootstrap resampling demonstrated consistent discrimination, the wide confidence intervals highlight the statistical uncertainty of these estimates.

Finally, the analytical framework proposed in this study was designed to prioritize clinical interpretability. While this approach enhances translational relevance, it may simplify complex immunologic interactions and requires validation in larger datasets to confirm robustness and reproducibility.

Despite these limitations, the study provides a proof-of-concept for the potential of integrating systemic immune markers into a clinically interpretable framework for patient-level stratification. This approach represents a shift from descriptive biomarker research toward actionable models that can support individualized care.

Future research should focus on external validation in larger and disease-specific cohorts, as well as on longitudinal studies assessing the predictive value of cytokine-based stratification for clinical outcomes. Integration with other data modalities, including imaging, endoscopy, and emerging digital tools, may further enhance the precision of disease monitoring strategies.

## 5. Conclusions

This exploratory study provides proof-of-concept that a simplified cytokine-based framework integrating interleukin-10 (IL-10) and interleukin-23 (IL-23) may enable the identification of clinically meaningful subgroups among patients with ulcerative colitis (UC) in endoscopic remission. By enabling stratification into low-, intermediate-, and high-risk categories for residual histologic activity, this approach offers a preliminary, clinically interpretable framework for individualized disease assessment. The absence of fecal calprotectin precludes evaluation of the incremental clinical value of this approach relative to established non-invasive biomarkers, and therefore, the proposed framework should be considered exploratory.

Importantly, the value of this model lies not in improving overall discrimination but in enhancing clinical interpretability and supporting risk-adapted decision-making. In this context, IL-10 functions as a sensitive detectability-based screening marker, while IL-23 may refine classification by identifying a small subgroup with a high observed proportion of persistent microscopic inflammation. However, estimates for this subgroup were based on only three patients and should therefore be interpreted cautiously.

These findings highlight the limitations of relying solely on endoscopic remission as a surrogate for disease control and support the potential complementary integration of immune-based biomarkers into monitoring strategies. The proposed framework may potentially contribute to guiding decisions regarding the need for histologic evaluation, closer follow-up, or therapeutic optimization; however, these implications remain hypothesis-generating and require prospective validation.

Further validation in larger, multicenter, disease-specific, and longitudinal studies is required to confirm the clinical utility and predictive value of this approach, as well as its integration with established biomarkers and emerging data-driven tools.

## Figures and Tables

**Table 1:** Baseline characteristics by endoscopic–histologic phenotype.

Variable	Concordant Remission (*n* = 22)	Discordant (*n* = 14)	Concordant Active Disease (*n* = 21)	*p*-Value
Age, years	49.0 (39.8–55.8)	46.0 (40.2–55.8)	56.0 (32.0–64.0)	0.481
Disease duration, years	5.0 (2.2–6.8)	6.5 (4.2–10.8)	4.0 (2.0–10.0)	0.282
CRP, mg/L	0.10 (0.10–1.18)	0.50 (0.10–1.95)	6.50 (1.40–10.80)	0.002
IL-10 (LOD-adjusted), pg/mL	4.75 (4.05–5.40)	1.95 (1.95–1.95)	1.95 (1.95–1.95)	<0.001
IL-23 ≥ 16.3 pg/mL, *n* (%)	0 (0.0%)	3 (21.4%)	3 (14.3%)	0.041
Hemoglobin, g/dL	13.75 (12.90–14.90)	13.60 (12.40–14.90)	13.20 (12.00–14.70)	0.552
WBC, ×10^9^/L	7.06 (6.24–7.72)	6.13 (5.34–7.54)	7.69 (5.38–9.20)	0.517
Male sex, *n* (%)	16 (72.7%)	5 (35.7%)	13 (61.9%)	0.085
Ulcerative colitis, *n* (%)	18 (81.8%)	13 (92.9%)	12 (57.1%)	0.037
Crohn’s disease, *n* (%)	4 (18.2%)	1 (7.1%)	9 (42.9%)	0.037
Current smoking, *n* (%)	5 (22.7%)	1 (7.1%)	4 (19.0%)	0.475

Footnote: Continuous variables are presented as median (interquartile range, IQR) and categorical variables as counts and percentages. Comparisons across groups were performed using the Kruskal–Wallis test for continuous variables and the chi-square or Fisher’s exact test for categorical variables, as described in the Section 2.11 Statistical Analysis. Median IL-10 values of 1.95 pg/mL correspond to the imputed LOD/2 value (half of the assay lower limit of detection of 3.9 pg/mL), indicating that more than half of patients within the respective groups had IL-10 concentrations below the assay detection threshold.

**Table 2:** Baseline clinical, laboratory, and biomarker characteristics stratified by sex.

Variable	Female (*n* = 25)	Male (*n* = 34)	*p*-Value
Age, years	54.0 (44.5–69.0)	49.0 (31.8–57.0)	0.174
Disease duration, years	6.0 (3.0–10.0)	4.5 (2.0–8.5)	0.264
CRP, mg/L	2.3 (0.1–9.8)	0.6 (0.1–6.1)	0.209
IL-10, pg/mL	2.8 (2.1–3.8)	3.5 (2.8–4.3)	0.155
IL-23, pg/mL	5.7 (3.5–13.4)	3.7 (1.3–10.1)	0.059
Hemoglobin, g/dL	12.7 (11.7–13.7)	14.0 (13.2–15.8)	0.001
WBC, ×10^9^/L	7.3 (5.9–8.5)	7.0 (5.4–8.0)	0.565

Footnote: Continuous variables are presented as median (interquartile range, IQR). Comparisons between female and male patients were performed using the Mann–Whitney U test. These analyses are exploratory and should be interpreted with caution due to the limited sample size and limited statistical power for robust sex-specific inference.

**Table 3:** Diagnostic performance and ROC-derived cut-off values for detection of histologic activity in patients with ulcerative colitis (UC) in endoscopic remission.

Biomarker	Cut-Off	Sensitivity (%)	Specificity (%)	AUC (95% CI)
IL-10 (LOD-adjusted)	<3.9 pg/mL	84.6%	77.8%	0.850 (0.704–0.996)
IL-10 (raw extrapolated values)	<3.9 pg/mL	92.3%	77.8%	0.906 (0.789–1.000)
IL-23 (detectable)	≥16.3 pg/mL	23.1%	94.4%	0.615 (0.402–0.829)

Footnote: Receiver operating characteristic (ROC) curve analysis was performed in patients with ulcerative colitis (UC) in endoscopic remission to evaluate the diagnostic performance of each biomarker for detecting histologic activity. Optimal cut-off values were determined using the Youden index. Sensitivity and specificity were calculated based on the selected cut-off values. IL-10 concentrations were analyzed using assay-reported values for primary modeling, while values below the limit of detection (LOD) were imputed as LOD/2 for descriptive purposes. A sensitivity ROC analysis was additionally performed using raw assay-reported extrapolated IL-10 values without LOD/2 substitution to evaluate the potential impact of left-censored data handling on biomarker performance. Given the observed distribution, IL-10 effectively functioned as a threshold-based biomarker in this cohort.

**Table 4:** Personalized cytokine-based risk stratification in patients with ulcerative colitis.

Risk Subgroup	Definition	*n*	Histologic Activity, *n* (%)
Low risk	IL-10 ≥ 3.9 pg/mL and IL-23 < 16.3 pg/mL	14	1 (7.1%)
Intermediate A	IL-10 ≥ 3.9 pg/mL and IL-23 ≥ 16.3 pg/mL	2	1 (50.0%)
Intermediate B	IL-10 < 3.9 pg/mL and IL-23 < 16.3 pg/mL	12	9 (75.0%)
High risk	IL-10 < 3.9 pg/mL and IL-23 ≥ 16.3 pg/mL	3	3/3 (100%; 95% CI 29.2–100%)

Footnote: Primary analysis restricted to patients with ulcerative colitis. Patients were stratified according to predefined IL-10 and IL-23 thresholds. The intermediate-risk group was further subdivided to explore heterogeneity in cytokine profiles. Given the limited sample size, particularly in the IL-23 detectable subgroup, results should be interpreted as exploratory. The high-risk subgroup consisted of only three patients.

**Table 8:** Sensitivity analysis using the stricter Geboes ≥ 2B.1 definition of histologic activity in patients with ulcerative colitis (UC) in endoscopic remission.

Parameter	Primary Definition (Geboes ≥ 2.0)	Sensitivity Definition (Geboes ≥ 2B.1)
Patients classified with histologic activity	14/31 (45.2%)	14/31 (45.2%)
Patients classified with histologic remission	17/31 (54.8%)	17/31 (54.8%)
Patient reclassification after applying stricter threshold	0/31 (0.0%)	No reclassification observed
IL-10 AUC (LOD-adjusted)	0.850	0.850
IL-10 AUC (raw assay-reported values)	0.906	0.906
IL-23 AUC	0.615	0.615
CRP AUC	0.581	0.581
Combined IL-10 + IL-23 model AUC	0.855	0.855
Cytokine-based risk subgroup classification	Identical subgroup distribution	Identical subgroup distribution

Footnote: A sensitivity analysis was performed using the stricter histologic activity threshold corresponding to neutrophilic inflammation (Geboes score ≥ 2B.1). No patient reclassification was identified compared with the predefined primary definition of Geboes ≥ 2.0, as all patients classified with histologic activity under the primary definition already demonstrated neutrophilic infiltration. Consequently, ROC performance estimates, multivariable model results, and cytokine-based risk subgroup allocation remained identical across both definitions.

## Data Availability

The datasets generated during the current study are available from the corresponding author on reasonable request. Data are not publicly available due to institutional and ethical restrictions.

## Supplementary Materials

The following supporting information can be downloaded at: https://www.mdpi.com/article/10.3390/jpm16050275/s1, Table S1: Distribution of biologic therapy exposure across ulcerative colitis (UC) endoscopic–histologic phenotype groups; Table S2: Exploratory ROC analysis of CRP for detection of histologic activity in patients with ulcerative colitis (UC) in endoscopic remission; Table S3: Exploratory pooled UC/CD cytokine-based risk stratification analysis in patients with endoscopic remission.

## Abbreviations

The following abbreviations are used in this manuscript:

IBD	Inflammatory bowel disease
UC	Ulcerative colitis
CD	Crohn’s disease
IL-10	Interleukin-10
IL-23	Interleukin-23
CRP	C-reactive protein
FC	Fecal calprotectin
ELISA	Enzyme-linked immunosorbent assay
ROC	Receiver operating characteristic
AUC	Area under the curve
LOD	Limit of detection
OR	Odds ratio
CI	Confidence interval
EPV	Events per variable
IQR	Interquartile range
SES-CD	Simple Endoscopic Score for Crohn’s Disease
WBC	White blood cell count
