# Supplementary material for: Personalized Risk Stratification of Residual Histologic Activity in IBD Using Circulating Cytokines
Source: J Pers Med. 2026 May 21;16(5):275. doi: 10.3390/jpm16050275 (PMC13208867; doi:10.3390/jpm16050275)
Supplement: Supplementary file 1 [file jpm-16-00275-s001.zip › jpm-4320555-supplementary.pdf]

Supplementary Table S1. Distribution of biologic therapy exposure across ulcerative colitis (UC) endoscopic–histologic phenotype groups

Phenotype group	Biologic therapy	Non biologic therapy
Concordant remission (n=18)	5/18 (27.8%)	13/18 (72.2%)
Discordant disease (n=13)	5/13 (38.5%)	8/13 (61.5%)
Concordant active disease (n=12)	5/12 (41.7%)	7/12 (58.3%)

Overall comparison: χ^2 p = 0.699

Footnote:Current therapy was summarized by phenotype group in the UC cohort. Advanced biologic therapy and combination therapy were grouped as biologic therapy, whereas 5-ASA and no treatment were grouped as no biologic therapy. Treatment distribution did not differ significantly across UC phenotype groups (χ^2 p = 0.699), suggesting that the observed cytokine-based stratification was not clearly explained by imbalance in biologic treatment exposure.

Supplementary Table S2. Exploratory ROC analysis of CRP for detection of histologic activity in patients with ulcerative colitis (UC) in endoscopic remission

Biomarker	Cut-off	Sensitivity (%)	Specificity (%)	AUC (95% CI)
CRP	≥14.7 mg/L	38.5%	72.2%	0.581 (0.364–0.798)

Footnote: The CRP cut-off identified in this study was substantially higher than commonly used clinical thresholds, reflecting the data-driven nature of ROC-derived cut-offs in a small cohort and resulting in limited clinical applicability.

Supplementary Table S3.Exploratory pooled UC/CD cytokine-based risk stratification analysis in patients with endoscopic remission

Risk group	Definition	n	Histologic activity, n (%)	p-value

Low risk	IL-10 ≥ 3.9 pg/mL and IL-23 < 16.3 pg/mL	18	1 (5.6%)	
Intermediate risk	IL-10 ≥ 3.9 pg/mL and IL-23 ≥ 16.3 pg/mL **or** IL-10 < 3.9 pg/mL and IL-23 < 16.3 pg/mL	15	10 (66.7%)	
High risk	IL-10 < 3.9 pg/mL and IL-23 ≥ 16.3 pg/mL	3	3 (100%)	**0.00012**

Overall comparison: Fisher's exact test, **p = 0.00012**.

Footnote: Patients were stratified into risk groups based on predefined IL-10 and IL-23 thresholds derived from ROC analysis.Associations between risk groups and histologic activity were evaluated using Fisher's exact test due to small expected cell counts.The observed proportion in the high-risk group is based on a very small number of patients (n = 3) and should be interpreted with caution.
